# Inverse Association of N-Terminal Pro-B-Type Natriuretic Peptide with Metabolic Syndrome in Patients with Congestive Heart Failure

**DOI:** 10.1371/journal.pone.0079096

**Published:** 2013-11-12

**Authors:** Huai-Ren Chang, Jen-Che Hsieh, Bang-Gee Hsu, Ling-Yi Wang, Michael Yu-Chih Chen, Ji-Hung Wang

**Affiliations:** 1 Division of Cardiology, Department of Internal Medicine, Buddhist Tzu Chi General Hospital, Hualien, Taiwan; 2 Division of Nephrology, Department of Internal Medicine, Buddhist Tzu Chi General Hospital, Hualien, Taiwan; 3 Institute of Medical Sciences, Tzu Chi University, Hualien, Taiwan; 4 School of Medicine, Tzu Chi University, Hualien, Taiwan; 5 Institute of Epidemiology & Preventive Medicine, College of Pulbic Health, National Taiwan University, Taipei, Taiwan; University of Otago, New Zealand

## Abstract

**Background:**

Metabolic syndrome has been shown to be associated with lower levels of plasma N-terminal pro-B-type natriuretic peptide (Nt-proBNP) in the general population. We sought to elucidate the relationship between Nt-proBNP and components of metabolic syndrome in patients with congestive heart failure (CHF).

**Methods:**

Fasting blood samples were obtained from 93 patients in our institution. Plasma levels of Nt-proBNP and other biochemical data were measured. The New York Heart Association (NYHA) classification system (I-IV) was used to define the functional capacity of CHF. Metabolic syndrome and its components were defined using diagnostic criteria from the International Diabetes Federation.

**Results:**

Forty-nine patients (52.7%) had CHF. There was a positive correlation between plasma Nt-proBNP levels and NYHA functional capacity in CHF patients. Plasma Nt-proBNP levels increased significantly with each increasing NYHA class of the disease. The prevalence of metabolic syndrome in CHF patients was higher than that in patients without CHF. Most importantly, we found that plasma Nt-proBNP levels were lower in CHF patients with metabolic syndrome attributable to inverse relationships between plasma Nt-proBNP and body mass index (β = −0.297), plasma triglyceride (β = −0.286) and homeostasis model assessment of insulin resistance (HOMA-IR; β = −0.346). Fasting glucose to insulin ratio (FGIR, an insulin sensitivity index) was positively associated with plasma Nt-proBNP levels (β = 0.491), and was the independent predictor of plasma Nt-proBNP levels in CHF patients.

**Conclusions:**

Plasma Nt-proBNP levels are inversely associated with metabolic syndrome in CHF patients. Reduced plasma Nt-proBNP levels in CHF patients may lead to impaired lipolysis and metabolic function, and may contribute to the development of metabolic syndrome in CHF patients.

## Introduction

B-type natriuretic peptide (BNP) is synthesized in the cardiomyocyte as a response to increased wall stress in relation to heart failure or myocardial ischemia [1]. BNP is produced initially as a 134 amino acid pre-pro-peptide, which is cleaved into proBNP, a 108 amino acid precursor molecule stored in secretory granules in the cardiomyocyte. Upon release, proBNP is cleaved by furin (a protease) into BNP (a 32 amino acid biologically-active protein) and N-terminal proBNP (Nt-proBNP, a 76 amino acid biologically-inert protein). High levels of BNP as well as Nt-proBNP are now promising cardiovascular risk markers and have been associated with albuminuria, hypertension, and left ventricular hypertrophy [2], [3]. Also, plasma Nt-proBNP level has been well known to rise in patients with congestive heart failure (CHF) and has become a powerful maker for diagnosis of CHF [4], [5]. On the other hand, it has also been hypothesized that a reduced natriuretic peptide response, called a natriuretic handicap, contributes to the increased susceptibility of obese individuals to fluid retention and heart failure [6].

CHF is a complex clinical syndrome that can result from any structural or functional cardiac disorders, including coronary artery disease, hypertensive heart disease, myocardial disease and valvular heart disease. Metabolic syndrome is a pathological condition with clustering of metabolic components, including glucose intolerance, elevated blood pressure, elevated triglyceride levels, low high-density lipoprotein-cholesterol (HDL-C) levels and obesity [7], [8]. Although the clinical utility of this designation is controversial, there is widespread agreement that it describes a subgroup of individuals with a high risk of cardiovascular disease [7]. The prevalence of metabolic syndrome in CHF patients is more than double compared with the general population in Japanese and suggests that the metabolic components may have a substantial effect on the development of both ischemic and non-ischemic CHF [9].

It has been shown that metabolic syndrome is associated with lower levels of Nt-proBNP in the general population [3], [10], [11]. Also, low Nt-proBNP levels have been noted in patients with obesity, especially visceral adiposity [12] and in obese patients with elevated left ventricular end diastolic pressure [13]. However, Nt-proBNP levels are not lower in obese patients with diabetes compared to obese patients without diabetes [14]. Furthermore, some recent prospective studies indicated that intensive lifestyle intervention could increase Nt-proBNP levels in obese diabetic patients [15], and baseline low Nt-proBNP levels could predict newly incident diabetes [16]. Accordingly, BNP and Nt-proBNP may play a substantial role in terms of whole-body and cardiac metabolism. Therefore, it is interesting to investigate the association between plasma Nt-proBNP levels and metabolic cardiovascular risk factors or metabolic syndrome in CHF patients. Our results showed that plasma Nt-proBNP levels were lower in CHF patients with metabolic syndrome attributable to inverse relationships between plasma Nt-proBNP and body mass index (BMI), plasma triglyceride and homeostasis model assessment of insulin resistance (HOMA-IR). Fasting glucose to insulin ratio (FGIR, i.e., an insulin sensitivity index) was positively associated with plasma Nt-proBNP levels and was the independent predictor of the plasma Nt-proBNP levels in CHF patients.

## Methods

### Patients

This prospective study was conducted in our institution during October 1st to December 31st, 2009. Ninety-three consecutive clinic patients, who had regularly followed up at the cardiology outpatient department every three months, were enrolled into this study (40 males and 53 females, age range 37–88 years). All participants were ambulatory patients, and their estimated daily activities exceeded three metabolic equivalents. Patients were excluded if they had any acute infection, acute myocardial infarction, pulmonary edema, chronic renal failure (serum creatinine ≥1.2 mg/dL) at the time of blood sampling or if they refused to provide informed consent for the study. This study was approved by the Protection of the Human Subjects Institutional Review Board of Buddhist Tzu Chi General Hospital. All participants had signed the written informed consent to participate in this study. The ethics committee also approved our consent procedure.

### CHF Classification

The New York Heart Association (NYHA) classification system (I, II, III, IV) was used to define the functional capacity of congestive heart failure, and the stage (A, B, C, D) of congestive heart failure was based on the American College of Cardiology Foundation and the American Heart Association 2005 guidelines [17]. The NYHA classification increases in severity from Class I to Class IV.

### Anthropometric Analysis

Body weight was measured in light clothing and without shoes to the nearest half-kilogram. Height was measured to the nearest 0.5 cm. Waist circumference was measured to the nearest 0.5 cm at the shortest point below the lower rib margin and the iliac crest. The BMI was calculated as weight (kilograms) divided by height squared (meters). Bioimpedance measurements of fat mass were performed according to the standard, tetrapolar, whole body (hand-foot) technique, using a single-frequency (50-kHz) analyzer (Biodynamic-450, Biodynamics Corporation, Seattle, MA, USA). Measurements were carried out by the same operator; fat mass was collected and analyzed by specific formulas provided by the manufacturer.

### Biochemical Investigations

Fasting blood samples of approximately 10 ml for measuring complete blood count (Sysmex K-1000, Bohemia, NY, USA) and other factors were immediately centrifuged at 3000 *g* for 10 min. Serum levels of blood urea nitrogen, creatinine, fasting glucose, total cholesterol, triglyceride, high-density lipoprotein-cholesterol (HDL-C), low-density lipoprotein-cholesterol (LDL-C), albumin, globulin, and C-reactive protein (CRP) were obtained and measured using an autoanalyzer (COBAS Integra 800, Roche Diagnostics, Basel, Switzerland) by our central laboratory in our institution.

### Nt-proBNP Measurements

Fasting plasma Nt-proBNP levels were determined with an Elecsys 20.10 bench top analyzer (Roche Diagnostics) with proBNP reagent pack (Roche Diagnostics) [18]. The analytical range extended from 20 pg/mL to 35,000 pg/mL. The intraassay coefficient of variation was 2.5% for a concentration of 175 pg/mL and 2% for a concentration of 1,070 pg/mL; the interassay coefficient of variation was 3.2% and 2.7%, respectively.

### Assessment of Insulin Resistance

Serum insulin levels were measured using the microparticle enzyme immunosobent assay (MEIA) method by an autoanalyzer (Abbott Laboratories, Abbott Park, IL, USA). Insulin resistance was estimated by the homeostasis model assessment of insulin resistance (HOMA-IR), calculated as fasting plasma glucose (mg/dL)×fasting serum insulin (µU/mL)/405. Pancreatic β-cell function was estimated by homeostasis model assessment of β-cell function (HOMA-β), calculated as fasting serum insulin (µU/mL)×360/fasting plasma glucose (mg/dL) −63 [19]. Also, we use fasting glucose to insulin ratio (FGIR) as an index of insulin sensitivity [20].

### Metabolic Syndrome Definition

The prevalence of metabolic syndrome was defined using the International Diabetes Federation definition [21]. People were classified as having metabolic syndrome if they had central (abdominal) obesity with a waist circumference ≥90 cm (men) or ≥80 cm (women) (Taiwanese criteria), and were matching two or more of the following criteria: (1) fasting triglycerides ≥150 mg/dL or use of fibrates or nicotinic acid; (2) reduced HDL-C (<40 mg/dL in men, <50 mg/dL in women); (3) systolic blood pressure ≥130 mmHg, diastolic blood pressure ≥85 mmHg or use of antihypertrnsive medications; and (5) fasting glucose ≥100 mg/dL or use of hypoglycemic medications. Type 2 diabetes was determined according to World Health Organization criteria [22]. A person was regarded as diabetes if the fasting glucose was either 126 mg/dL or more, or if the 2 h glucose during an oral glucose tolerance test was 200 mg/dL or more, or if he/she was using hypoglycemic medications.

### Statistical Analyses

Data were expressed as means ± standard deviation (SD) and were tested for normal distribution by Kolmogorov-Smirnov statistics. Categorical variables were analyzed by the Chi-square test. Comparisons between patients were performed using Student’s independent *t* test (two-tailed) for normally distributed data or the Mann-Whitney U test for parameters that presented with non-normal distribution (e.g., fasting glucose, CRP, Nt-proBNP). The significance of differences of Nt-proBNP between groups (NYHA functional CHF classes) was analyzed by the Kruskal-Wallis analysis of variance (AVONA) test. Clinical variables that correlated with plasma Nt-proBNP levels in CHF patients were evaluated by univariable linear regression analysis. Variables that were significantly associated with Nt-proBNP in CHF patients were tested for independency by multivariable linear regression analysis. Data were analyzed using SPSS for Windows (version 13.0; SPSS Inc., Chicago, IL, USA). A *p* value of less than 0.05 was considered statistically significant.

## Results

**Figure 1:**
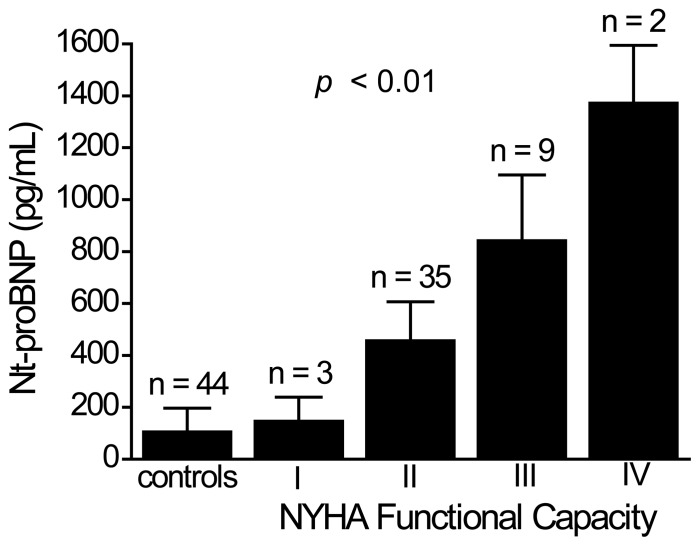
The association between plasma Nt-proBNP levels and NYHA functional capacity in the 49 CHF patients. Data was analyzed by the Kruskal-Wallis analysis of variance (AVONA) test.

The clinical characteristics of total 93 patients with or without CHF are shown in Table 1. Forty-nine patients (52.7%) had CHF (including 32 dilated cardiomyopathy, 12 ischemic heart disease and 5 valvular heart disease). Patients with CHF had older age (66.7±11.2 years v.s. 62.5±7.8 years, *p* = 0.037) and higher plasma Nt-proBNP levels (495.4±142.9 pg/mL v.s. 109.1±88.3 pg/mL, *p*<0.001) than patients without CHF. In addition, there was no difference of other clinical variables (e.g., height, body weight, BMI, CRP, total cholesterol, triglyceride, HDL-C, LDL-C, HOMA-IR, et al.) between patients with and without CHF (Table 1). According to NYHA functional classification, there were 3, 35, 9 and 2 CHF patients in NYHA class I, II, III and IV, respectively. There was a positive correlation between plasma Nt-proBNP levels and NYHA functional capacity in CHF patients. Also, plasma Nt-proBNP levels increased significantly with each increasing NYHA class of the disease (Figure 1). In CHF patients with NYHA class I, plasma Nt-proBNP levels were still higher than those without CHF (158.1±89.4 pg/mL v.s. 109.1±88.3 pg/mL, Figure 1).

**Table 1:** Clinical variables of the patients with or without congestive heart failure.

Items	No CHF (n = 44)	CHF (n = 49)	*p* value
Age (year)	62.5±7.8	66.7±11.2	0.037*
Height (cm)	158.4±7.0	160.1±7.7	0.268
Body weight (kg)	71.6±10.5	72.4±13.5	0.761
Waist circumference (cm)	95.1±8.7	97.0±10.3	0.384
Body mass index (BMI; kg/m^2^)	28.6±3.8	28.1±3.9	0.547
Body fat mass (%)	36.2±7.4	36.9±6.9	0.634
Body water (%)	34.0±5.6	34.0±6.7	0.994
White blood count (x1000/uL)	7.0±2.0	6.9±1.8	0.805
Hemoglobulin (g/dL)	13.6±1.8	13.9±1.7	0.549
Albumin (g/dL)	4.5±0.8	4.4±0.2	0.241
Globulin (g/dL)	2.9±0.5	2.9±0.4	0.608
Total cholesterol (mg/dL)	191.7±43.6	192.8±34.0	0.886
Triglyceride (mg/dL)	150.8±84.2	161.1±95.4	0.585
HDL-C (mg/dL)	48.1±13.9	45.5±12.7	0.365
LDL-C (mg/dL)	127.0±43.4	124.9±29.8	0.788
Fasting glucose (mg/dL)	117.7±34.8	123.5±69.9	0.590
Blood urea nitrogen (mg/dL)	16.2±4.6	17.2±5.0	0.310
Creatinine (mg/dL)	0.81±0.20	0.85±0.18	0.202
CRP (mg/dL)	0.28±0.26	0.40±0.59	0.972
Systolic pressure (mmHg)	130.9±7.3	131.4±11.3	0.819
Diastolic pressure (mmHg)	74.5±8.1	75.1±7.6	0.730
Insulin (µU/dL)	11.22±10.85	14.22±23.98	0.426
HOMA-IR	3.08±2.59	4.07±5.51	0.453
HOMA-β	115.6±165.5	146.5±347.9	0.453
FGIR	19.4±18.5	16.7±14.9	0.650
**NT-pro BNP (pg/mL)**	109.1±88.3	495.4±142.9	<0.001*

CHF, congestive heart failure; HDL-C, high-density lipoprotein-cholesterol; LDL-C, low-density lipoprotein-cholesterol; CRP, C-reactive protein; HOMA-IR, homeostasis model assessment of insulin resistance; HOMA-β, homeostasis model assessment of β cell function; FGIR, fasting glucose to insulin ratio.

* *p*<0.05 was considered statistically significant after performing the Student *t*-test or Mann-Whitney U test (Nt-proBNP, CRP, fasting glucose, insulin, HOMA-IR, HOMA-β, FGIR).

In all 49 CHF patients, 23 patents (46.9%) met criteria for the metabolic syndrome, whereas the remaining 26 patients (53.1%) did not. In contrast, there were only 6 patients (13.6%) with metabolic syndrome in all 44 patients without CHF. The prevalence of metabolic syndrome in CHF patients was thus more than triple compared with patients without CHF. Furthermore, the CHF patients with metabolic syndrome had lower plasma Nt-proBNP levels than those without metabolic syndrome (356.1±127.8 pg/mL v.s. 653.1±214.5 pg/mL, *p* = 0.042). These results demonostrated that plasma Nt-proBNP levels were inversely associated with metabolic syndrome in CHF patients.

The results of univariable linear regression between plasma Nt-proBNP levels and clinical variables in the CHF patients are shown in Table 2. Height (β = −0.301, *p* = 0.036), body weight (β = −0.359, *p* = 0.011), BMI (β = −2.94, *p* = 0.038), triglyceride (β = −0.286, *p* = 0.046), and HOMA-IR (β = −0.346, *p* = 0.015) were negatively correlated with plasma Nt-proBNP levels among the CHF patients. On the other hand, FGIR (β = 0.491, *p*<0.001) was positively correlated with plasma Nt-proBNP levels among the CHF patients. We then put these six significant variables together and performed a multivariable linear regression analysis. The result showed that FGIR (β = 0.491, R square = 0.241, *p*<0.001) was the independent predictor of plasma Nt-proBNP levels (Table 3).

**Table 2:** Correlation of plasma Nt-proBNP levels and clinical variables by univariable linear regression analyses among the 49 congestive heart failure patients.

Items	β	*p* value
Age (year)	−0.097	0.509
Height (cm)	−0.301	0.036*
Body weight (kg)	−0.359	0.011*
Waist circumference (cm)	−0.294	0.063
Body mass index (BMI; kg/m^2^)	−0.297	0.038*
Body fat mass (%)	−0.068	0.650
White blood count (x1000/uL)	−0.127	0.410
Hemoglobulin (g/dL)	−0.143	0.355
Albumin (g/dL)	−0.160	0.272
Globulin (g/dL)	0.037	0.802
Total cholesterol (mg/dL)	−0.052	0.723
Triglyceride (mg/dL)	−0.286	0.046*
HDL-C (mg/dL)	0.128	0.382
LDL-C (mg/dL)	0.138	0.343
Fasting glucose (mg/dL)	0.242	0.093
Blood urea nitrogen (mg/dL)	0.124	0.396
Creatinine (mg/dL)	−0.043	0.767
CRP (mg/dL)	−0.062	0.671
Insulin (µU/dL)	−0.263	0.068
HOMA-IR	−0.346	0.015*
HOMA-β	−0.197	0.174
FGIR	0.491	<0.001*

HDL-C, high-density lipoprotein-cholesterol; LDL-C, low-density lipoprotein-cholesterol; CRP, C-reactive protein; HOMA-IR, homeostasis model assessment of insulin resistance; HOMA-β, homeostasis model assessment of β cell function; FGIR, fasting glucose to insulin ratio.

* *p*<0.05 was considered statistically significant after univariate linear analyses.

**Table 3:** Multivariable stepwise linear regression analysis of height, body weight, body mass index, triglyceride, HOMA-IR and FGIR: correlation to plasma Nt-proBNP levels among 49 congestive heart failure patients.

Items	β	R square	*p* value
FGIR	0.491	0.241	<0.001*

HOMA-IR, homeostasis model assessment of insulin resistance; FGIR, fasting glucose to insulin ratio.

* *p*<0.05 was considered statistically significant after multivariate stepwise linear regression analyses.

## Discussion

The principal findings of this study are that plasma Nt-proBNP levels are lower in CHF patients with metabolic syndrome attributable to inverse relationships between plasma Nt-proBNP and BMI, plasma triglyceride and insulin resistance (HOMA-IR). Insulin sensitivity index (i.e., FGIR) is positively associated with plasma Nt-proBNP levels and is the independent predictor of the plasma Nt-proBNP levels in CHF patients.

### Positive Correlation between Plasma Nt-proBNP Level and NYHA Functional Capacity in CHF Patients

The prevalence of CHF increases with age and is expected to rise as a result of the increased life expectancy in communities [23]. Our results also show that CHF patients have older age than patients without CHF. Moreover, our patients with CHF have higher plasma Nt-proBNP levels compared to those without CHF. There is a positive correlation between plasma Nt-proBNP levels and NYHA functional capacity in CHF patients. Also, plasma Nt-proBNP levels significantly increase with each increasing NYHA class of the disease. In CHF patients with NYHA class I, plasma Nt-proBNP levels are still higher than those without CHF. Both of this study and one previous study [5] suggest that the severity of CHF could be determined on the basis of plasma Nt-proBNP levels. Plasma Nt-proBNP levels have been well known to rise in CHF patients [4], [5]. However, further studies with larger sample sizes may help to determine the reference range for plasma Nt-proBNP levels for the corresponding functional class of CHF.

BNP and Nt-proBNP are well known to derive from a common precursor (proBNP). The half-life of BNP is only 18 minutes due to active clearance from the circulation via natriuretic peptide receptors and degradation by neutral endopeptidases in the blood stream [24]. BNP levels are not stable *in vitro* for a long period and drop significantly over the first 24 hours following collection [25]. Also, if blood is collected into glass tube, BNP levels may fall due to activation of the kallikrein system. In contrast, Nt-proBNP is not biological active and dose not have active clearance mechanisms. The half-life of Nt-proBNP is approximately 60–120 minutes. Therefore, Nt-proBNP is dramatically more stable than BNP, with very little variation after collection for at least 72 hours. In addition, Nt-proBNP may be collected into glass tube without any degradation. Thus, Nt-proBNP assay may be more sensitive than BNP in the certain scenario [24], [25], [26]. Accordingly, we measured Nt-proBNP levels but not BNP levels in this study.

### Inverse Association of Nt-proBNP with Metabolic Syndrome in CHF Patients

Obese individuals in the cohorts of the Framingham Heart Study were found to have lower plasma BNP levels than those with normal weight [2]. An inverse relationship between plasma BNP and BMI has also been described recently [2], [27], and has been hypothesized to be a potential link between obesity and hypertension because obese people may have lower natriuretic peptides [2]. Our study also shows that body weight and BMI are negatively associated with plasma Nt-proBNP levels in CHF patients. Lower plasma BNP levels have been associated with the development of insulin resistance in normal population [3]. In addition to body weight and BMI, our results also demonstrate that plasma triglyceride and HOMA-IR are both negatively associated with plasma Nt-proBNP levels among CHF patients. Furthermore, FGIR (an insulin sensitivity index) is positively associated with plasma Nt-proBNP levels, and is the independent predictor of plasma Nt-proBNP levels in CHF patients. In summary, there is an inverse association of Nt-proBNP with metabolic syndrome in patients with CHF.

### Nt-proBNP Linking the Heart and Adipose Tissue in CHF Patients

The heart and adipose tissue are both endocrine organs, and there is increasing evidence for cross talk between them, although precise mechanisms remain unclear. Of particular importance is the role that such cross talk could play in both total body metabolism and cardiac metabolism [28], [29]. Our results demonstrate that the prevalence of metabolic syndrome in CHF patients is significantly higher than that in patients without CHF (46.9% v.s. 13.6%, respectively, *p*<0.05), suggesting that metabolic components may have a substantial effect on the development of CHF. One recent large registry study in Japan also has shown that the prevalence of metabolic syndrome in CHF patients is more than double compared with the general population [9]. These evidences propose that the fact of elevated Nt-proBNP in CHF patients may provide a protection to prevent the development of metabolic syndrome. In contrast, decreased Nt-proBNP levels would aggravate the prevalence of metabolic syndrome in CHF patients.

By now the cardiovascular community is well aware that atrial natriuretic peptide (ANP) and BNP promote vasodilation and natriuresis, improve diastolic function, suppress aldosterone, and inhibit cardiac hypertrophy and fibrosis [30], [31]. Less well known is that ANP and BNP have metabolic roles; specifically, ANP and BNP are lipolytic and slow gastric emptying and absorption [32], [33]. Indeed, the binding of ANP or BNP to the natriuretic peptide receptor-A (NPR-A), which is present in adipocytes, results in the production of the second messenger cGMP. The cGMP in turn activates protein kinase G, leading to phosphorylation of hormone sensitive lipase (HSL). The HSL is thus activated, and hydrolysis of fatty acids ultimately occurs [32], [34]. These lipolytic actions of ANP and BNP imply an emerging role for the heart in human metabolism [35]. Reduced natriuretic peptide signaling could have detrimental effects via the promotion of lipid accumulation in adipose tissue and skeletal muscle. Our results suggest that low levels of Nt-proBNP may lead to reduced lipolysis and excessive weight gain in CHF patients, which may be one of the biological alterations that contribute to the development of metabolic syndrome in CHF patients.

### Study Limitations

Our study has some limitations. First, this study was of cross-sectional design. Therefore, our results should be investigated in long-term prospective studies before a causal relationship between plasma Nt-proBNP and metabolic syndrome in CHF patients can be established. Second, most studies have focused on effects of BNP, but not Nt-proBNP, on lipolysis and adipose tissue. Further studies are needed to evaluate the Nt-proBNP effects on metabolic syndrome in CHF patients.

## Conclusion

Our results show that there is a positive correlation between plasma Nt-proBNP levels and NYHA functional capacity in CHF patients. Plasma Nt-proBNP levels increase significantly with each increasing NYHA class of the disease. Also, the prevalence of metabolic syndrome in CHF patients is higher than that in patients without CHF. Most importantly, we find that there is an inverse association of Nt-proBNP with metabolic syndrome in CHF patients. Also, FGIR is positively associated with plasma Nt-proBNP levels, and is the independent predictor of plasma Nt-proBNP level in CHF patients. Reduced plasma Nt-proBNP levels in CHF patients may lead to impaired lipid and glucose metabolism, and may contribute to the development of metabolic syndrome in CHF patients.
